# A58 ENTERIC TUFT CELLS MODULATE LOCAL BUT NOT SYSTEMIC HOST IMMUNE RESPONSES TO CO-ORDINATE TIMELY EXPULSION OF HYMENOLEPIS DIMINUTA

**DOI:** 10.1093/jcag/gwac036.058

**Published:** 2023-03-07

**Authors:** S Rajeev, S Li, K Flannigan, A Wang, D M McKay

**Affiliations:** 1 Gastrointestinal Research Group and Inflammation Research Network, Department of Physiology and Pharmacology, Snyder Institute for Chronic Diseases, Cumming School of Medicine; 2 Host-Parasite Interactions Program, University of Calgary, Calgary, Canada

## Abstract

**Background:**

The intestinal tuft cell is a versatile epithelial cell implicated in host detection and defence against enteric nematode and trematode parasites by producing interleukin(IL)-25 and cysteinyl leukotrienes. We have shown that immunocompetent mice develop small intestinal tuft cell hyperplasia during infection with the cestode, *Hymenolepis diminuta*. Whether tuft cells coordinate murine expulsion of *H. diminuta* and if this response is similar to other well-established parasitic models is unknown.

**Purpose:**

To test 1) if tuft cells coordinate host detection and immune responses to *H. diminuta*; and, 2) if the lack of functional B, T cells and innate lymphoid cells (ILC2s) affect tuft cell hyperplasia and expulsion of the parasite.

**Method:**

Male and female *pou2f3*^-/-^/^-/+^ littermates, *rag-1*^-/-^ and C57BL6 mice were infected with 5 cysticercoids of *H. diminuta*. ILC2s were depleted in *rag*-*1*^*-/*-^ with anti-CD90.2 (250μg/mouse, intraperitoneal). At necropsy, small intestines were flushed with cold PBS to count worms. IgG1, IgG2α and mast cell protease-1 (MCPT-1) was measured in serum and supernatants from splenic cells stimulated with ConA (48h) were assessed by ELISA for IL-4, -5, -10 and -13. Doublecortin-like kinase -1 (DCLK-1)+ tuft cells, goblet cells and eosinophils were identified in sections of paraffin embedded or cryopreserved mid-jejunum sections. mRNA of small intestinal epithelium enriched fractions isolated from mice at control and 11 days post-infection (dpi) was evaluated by qPCR.

**Result(s):**

Unlike *pou2f3*^+/-^ and C57Bl6 mice that expel *H. diminuta* by 8-11 dpi., *pou2f3*^-/-^ mice harbour *H. diminuta* at 11 dpi. *Pou2f3*^*-/*-^ mice show similar splenic IL-4, -5, -10, -13, and serum IgG1, IgG2a and MCPT-1 levels at 8 dpi. and higher splenic IL-4, -10 at 11 dpi. compared to *pou2f3*^*+/*-^ mice. In contrast, *pou2f3*^+/-^ mice show higher jejunal goblet cell hyperplasia at 8 dpi., higher levels of small intestinal epithelium expression of *dclk-1, alox5* and *il-25* and jejunal eosinophilia at 11 dpi. compared to *pou2f3*^*-/*-^ mice. At 11 dpi, ILC^depleted^*rag-1*^-/-^ develop tuft cell hyperplasia to the same extent as infected PBS/isotype controls and show no trace of intestinal worms in the lumen.

**Conclusion(s):**

Enteric tuft cells coordinate rapid expulsion of *H. diminuta* from mice, but are not essential for the host to detect and develop Th2 systemic responses in response to the worm. However, in the absence of tuft cells, deficiencies are observed in gut-specific effector immune events commonly associated with timely expulsion of enteric helminths; illustrating a disparity between local and systemic immunity following infection with this tapeworm parasite in a non permissive host. The lack of a functional adaptive immune system and surprisingly, depletion of ILC2s, does not completely abrogate worm expulsion or tuft cell hyperplasia in *rag-1*^*-/*-^ at 11 dpi. with *H. diminuta*. This suggests that an innate immune cell population other than CD90.2^+^ ILC2s amplifies Th2 immunity to *H. diminuta*-infection.

**Please acknowledge all funding agencies by checking the applicable boxes below:**

Other

**Please indicate your source of funding;:**

NSERC, , Eyes High doctoral scholarship, Alberta Graduate excellence scholarship

**Disclosure of Interest:**

None Declared

